# Dysregulation of Systemic Immunity in Aging and Dementia

**DOI:** 10.3389/fncel.2021.652111

**Published:** 2021-06-22

**Authors:** Jenny Lutshumba, Barbara S. Nikolajczyk, Adam D. Bachstetter

**Affiliations:** ^1^Spinal Cord and Brain Injury Research Center, University of Kentucky, Lexington, KY, United States; ^2^Department of Neuroscience, University of Kentucky, Lexington, KY, United States; ^3^Sanders-Brown Center on Aging, University of Kentucky, Lexington, KY, United States; ^4^Department of Pharmacology and Nutritional Science, University of Kentucky, Lexington, KY, United States; ^5^Barnstable Brown Diabetes and Obesity Center, University of Kentucky, Lexington, KY, United States

**Keywords:** monocytes, T cells, Treg, Th17, CD4, CD8, neuroimmunology, neuroinflammation

## Abstract

Neuroinflammation and the tissue-resident innate immune cells, the microglia, respond and contribute to neurodegenerative pathology. Although microglia have been the focus of work linking neuroinflammation and associated dementias like Alzheimer’s Disease, the inflammatory milieu of brain is a conglomerate of cross-talk amongst microglia, systemic immune cells and soluble mediators like cytokines. Age-related changes in the inflammatory profile at the levels of both the brain and periphery are largely orchestrated by immune system cells. Strong evidence indicates that both innate and adaptive immune cells, the latter including T cells and B cells, contribute to chronic neuroinflammation and thus dementia. Neurodegenerative hallmarks coupled with more traditional immune system stimuli like infection or injury likely combine to trigger and maintain persistent microglial and thus brain inflammation. This review summarizes age-related changes in immune cell function, with special emphasis on lymphocytes as a source of inflammation, and discusses how such changes may potentiate both systemic and central nervous system inflammation to culminate in dementia. We recap the understudied area of AD-associated changes in systemic lymphocytes in greater detail to provide a unifying perspective of inflammation-fueled dementia, with an eye toward evidence of two-way communication between the brain parenchyma and blood immune cells. We focused our review on human subjects studies, adding key data from animal models as relevant.

## Introduction

Dementia is a disease of the mind, but the whole body contributes to brain demise. The corollary is also likely true: the degeneration of the brain likely contributes to the demise of the body. Chronological age is the proverbial thumb on the scale, weighing down the body and the brain until their inevitable end.

As an umbrella term, dementia defines a group of disorders associated with the declining inability to think and remember (cognitive abilities) and negatively affects mood and emotion. Dementia robs a person of themself and impairs their ability to function independently. Alzheimer’s Disease (AD) is the most well-known and well-studied type of dementia, but it is only one of aging’s many neurodegenerative diseases (Kovacs, 2015, 2020; Neltner et al., 2016; Nelson et al., 2019; Karanth et al., 2020; McKee, 2020).

What has become apparent over the last couple of decades is that a pure AD-type of dementia – that is, one associated with only amyloid plaques and neurofibrillary tangles – is rare (Jellinger, 2020). It is true that AD pathological lesions are common and begin to develop even in middle age (Nelson et al., 2011). Most people who die over the age of 70 and are cognitively healthy for their age will have at least some amyloid plaques or neurofibrillary tangles (Price and Morris, 1999). Most individuals with profound cognitive decline will have a high burden of neuropathological changes, including amyloid plaques and neurofibrillary tangles, vascular, synucleinopathy, and TDP-43 pathology (Karanth et al., 2020). We also know that the vascular contribution to cognitive impairment and dementia (VCID) is profound, and here too, we can see the connection between the body and the brain (Ighodaro et al., 2017; Blevins et al., 2020; Zlokovic et al., 2020).

During life, clinical symptoms and biomarkers guide the physician to classifying a person’s dementia as a vascular-type or AD-type dementia, for example. Yet, disease confirmation requires postmortem neuropathological evaluation (Montine et al., 2012; Abner et al., 2017). This distinction is essential for our review. Much of the research we will discuss will be probable AD-type dementia. Few, if any, of the studies will have confirmed that AD neuropathological changes caused dementia. Moreover, the understanding of dementia is ever-evolving.

Neuroinflammation is a prominent feature and a likely contributor to neurodegenerative disease pathogenesis. However, AD and related dementias differ from autoimmune diseases, such as multiple sclerosis, in which T and B lymphocytes invade the central nervous system (CNS) parenchyma. Instead, in AD and related dementias, the brain-resident macrophages, microglia, appear to be the primary component of the immune system acting locally in the CNS tissue (Prinz and Priller, 2017). In healthy brain, parenchyma, lymphocytes, and blood-derived monocytes are absent or rare (Prinz and Priller, 2017). In addition, the CNS barriers establish immune privilege by tightly regulating the movement of cells and fluid into and out of the tissue (Engelhardt et al., 2017). Thus, the interaction of the CNS with the systemic immune system is profoundly different than other tissues.

While the involvement of blood-derived immune cells in CNS tissue is limited in AD and related dementias, it is well-known that a systemic immune response affects the brain. For instance, an immune response to systemic infection can cause delirium without immune cells attacking and killing neurons (Perry et al., 2007). Experimentally, the response to systemic inflammation causes a behavioral response (i.e., lethargy, anhedonia, decreased concentration) termed “sickness behavior” (Hart, 1988; Perry et al., 2007). Multiple direct routes foster communication and functional impacts of a systemic immune response to the CNS. For instance, cytokines and other inflammatory mediators in the blood can enter the brain via the circumventricular organs, or cross the blood–brain barrier (BBB), leading to increased reactive microglia (Perry et al., 2007; Erickson et al., 2020).

In addition to microglia, the brain endothelium is a major cellular target of systemic inflammation. For instance, brain endothelial cells express high levels of the receptor for one of the major proinflammatory cytokines, interleukin-1 (IL-1) (Liu and Quan, 2018; Liu et al., 2019). IL-1 is elevated in AD patients’ brains (Griffin et al., 1989). The brain endothelial cells also alter the permeability and transport of substances across the BBB (Erickson et al., 2020). Systemic inflammation can alter neurovascular coupling (Iadecola, 2017) and increase the adhesion of neutrophils to the brain capillaries, blocking blood flow (Cruz Hernandez et al., 2019). At the extreme, systemic inflammation also causes hypoxic/ischemic changes, including small and larger hemorrhagic infarcts (Thakur et al., 2021). Thus, it is easy to envision how systemic inflammation could synergize and further exacerbate VCID-related pathology in the elderly.

In older individuals, multiple systemic inflammatory events often precede and are hypothesized to contribute to downward spikes in cognitive function (Perry et al., 2007). Thus, while delirium and sickness behavior are transient, there is compelling evidence that systemic immune responses and systemic inflammation have lasting effects on the brain, particularly in older fragile individuals. Evidence for this conjecture comes from aged animals given a systemic immune challenge such as bacterial lipopolysaccharides (LPS) that have prolonged sickness behavior, increased cognitive impairment, and increased reactive microglia (Norden et al., 2015).

Individuals with AD also have higher circulating cytokine amounts compared to healthy controls. For example, across multiple studies, serum/plasma individuals with AD had more IL-1β, IL-6, IL-18, and TNFα than cognitively healthy subjects (Swardfager et al., 2010; Su et al., 2019). Presumably, the circulating cytokines are elevated from some ill-defined baseline and are not generated to counter an active infection or injury, but instead suggest a heightened state of basal inflammation.

Genetics, environment, and systemic diseases such as diabetes are underlying causes of a “primed” state of inflammation. Aging is also a significant driver of inflammation. In addition, nearly every aspect of the immune system is affected by age, collectively termed immunosenescence (Nikolich-Zugich, 2018). Therefore, as diseases of aging, AD, and related dementias must also consider the impact of the aged immune system on the disease.

This review evaluates the evidence for changes in the systemic immune system with age and dementia. We believe changes in the immune system may contribute to dementia, particularly when the immune system responds to disease, infection, or injury. Our central focus is on the understudied systemic cellular arm of the immune system. Aging is the inflammatory background common across dementias; thus, our review starts with an outline of age-related changes in the systemic immune system. We will then describe the sparse studies of systemic immune cell differences in AD that contrast with the extensive understanding of age-related inflammation. As the number of AD-related studies of the systemic immune system is limited, we will describe those studies in greater detail. While animal models are instrumental in testing mechanisms and therapeutics, we have focused our review on studies in people, as these studies can provide the most remarkable insight into the natural history of dementia.

## Immunesenescence Changes in People With Age

The progressive decline in immunity contributes to poor responsiveness to vaccines, increased incidences of infections, and decreased immunosurveillance of neoplasms amidst a chronic state of poorly defined inflammation. The decline of the immune system with age is not limited to one element due to interplay between the immune cells (both innate and adaptive), changes to microenvironment (including lymphoid and non-lymphoid tissues), and alterations in circulating inflammatory mediators (cytokines and chemokines, among other soluble factors) (Nikolich-Zugich, 2018). The immune system’s age-related changes are best described as dysregulated and not simplistically skewed pro- or anti-inflammatory as described in many excellent reviews of immunosenescence in general (High et al., 2012; Nikolich-Zugich, 2018; Velardi et al., 2020).

### Innate Immune Cell Changes With Age

Innate immunity and macrophage/microglia have traditionally taken center stage in CNS disorders (Bachstetter et al., 2015; Greenhalgh et al., 2020; Madore et al., 2020; Shahidehpour et al., 2021). Therefore, we will begin our discussion of immunosenescence with the innate immune system with a focus on neutrophils, dendritic cells (DCs), and monocytes.

Neutrophils are the first responder and act locally by migrating (chemotaxis) to the site of tissue injury or pathogen. Once the neutrophils leave the circulation by extravasation, they play essential roles in phagocytosis and killing the invading microbes. Neutrophils are short-lived in circulation, with a half-life of 8–12 h that is offset by production of large numbers (1–2 × 10^11^ neutrophils per day) (Lord et al., 2001). With advanced age, the number of circulating neutrophils in the blood is stable, but they proliferate less in response to G-CSF (Chatta et al., 1993; Born et al., 1995; Wenisch et al., 2000). Neutrophils in older individuals (66–93 years old) are still able to increase in number (neutrophilia) in response to a bacterial infection to reach a normal range (Lord et al., 2001). The neutrophil’s microbicidal function decreases with age, and includes reduced chemotaxis, reduced phagocytosis, decreases in neutrophil extracellular traps, and a slower resolution of inflammation (Shaw et al., 2010; Montgomery and Shaw, 2015).

To the best of our knowledge, it is not known in people if neutrophilia, chemotaxis, and extravasation are maintained in older individuals in response to a brain injury, such as a cerebral microhemorrhages. In response to lung injury in mice, neutrophils migrate to the lung similarly in aged and young adult animals (Nomellini et al., 2008). The neutrophil response resolved in the young adult animals by 24-h after the lung injury, but in aged animals, neutrophil numbers at 24 hrs post-injury were indistinguishable from the 6-h post-injury peak. Aged neutrophil response was resolved by blocking CXCR2 dependent chemokine signaling (MIP2 and KC), which did not otherwise adversely affect wound recovery. These data support the hypothesis that delayed resolution of the neutrophilic response in the brain of aged individuals after an injury may exacerbate the secondary damage caused by the injury.

**FIGURE 1:**
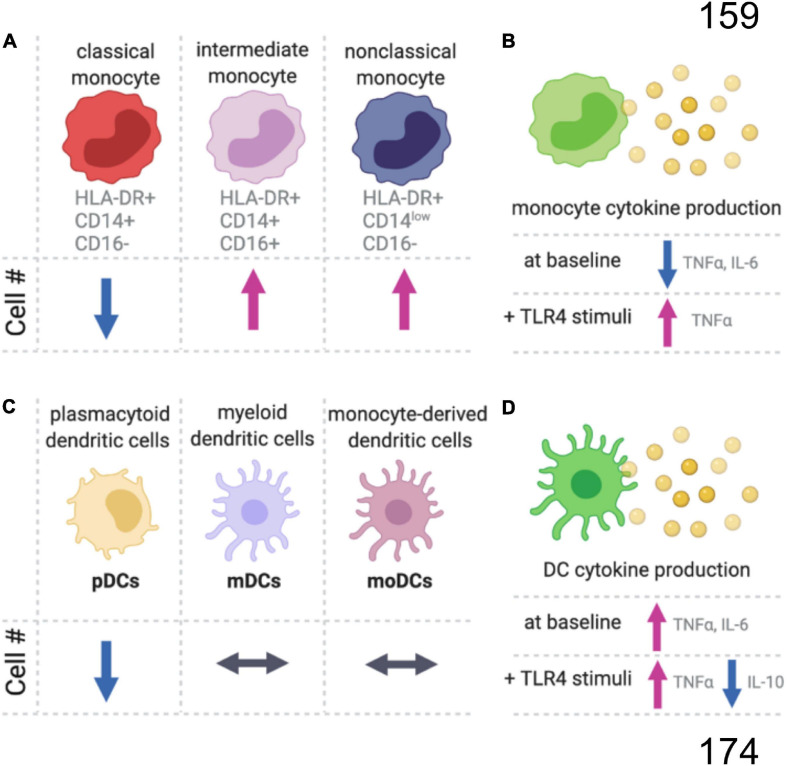
Summary of age-related changes in monocyte and dendritic cells. **(A)** In the blood of older aged people, there is a decrease in the proportion of classical monocytes, while the intermediate and non-classical monocyte populations are increasing compared to younger adults. **(B)** Monocytes also have an age-dependent decrease in proinflammatory cytokines at baseline, but with TLR4 stimulation (LPS) the aged monocytes produce more proinflammatory cytokines than monocytes from younger individuals. **(C)** There are age-related changes in subpopulations for dendritic cells (DC), and **(D)** the aged DCs produced more proinflammatory and less anti-inflammatory cytokines.

Monocytes are circulating leukocytes that originate in the bone marrow from a common myeloid progenitor cell. Monocytes account for around 5–10% of the circulating leukocytes in human blood (Guilliams et al., 2018). Monocytes and monocyte-derived macrophages are from a different cellular linage than most tissue-resident macrophages (i.e., microglia), which have an embryonic origin from yolk sac progenitors (Prinz et al., 2017). In humans, monocytes can be classified as classical HLA-DR^+^CD14^+^CD16^–^, intermediate HLA-DR^+^CD14^+^CD16^+^, and non-classical HLA-DR^+^CD14^*low*^CD16^+^ (Cros et al., 2010; Hearps et al., 2012; Guilliams et al., 2018; Figure 1). Classical monocytes are about 85% of all monocytes and remain in circulation for around 1 day as they traffic to tissues, such as the intestine, to repopulate the tissue-resident macrophages. Alternatively, classical monocytes can differentiate into non-classical monocytes, which can live for 7 days in humans. However, non-classical monocytes might develop without first becoming a classical monocyte (Guilliams et al., 2018).

The replacement of tissue macrophages by classical monocytes is not believed to occur in the CNS, at least not in the healthy brain (Waisman et al., 2015; Herz et al., 2017). However, much of what we know about the turnover of tissue macrophages is from rodent studies. There is only limited understanding of microglia replacement in people by monocyte-derived macrophages and the influence of aging, injury, and disease on these processes in people is not well described. The non-classical monocytes have been found to have important functions in patrolling the vasculature, including the arterial, capillary, and venous compartments (Guilliams et al., 2018), and this could be a mechanism for how monocytes might sense changes to the cerebral vasculature as part of VCID.

The number of monocytes increases linearly with age and comparing the number of monocytes in 20- to 30-year-old individuals to the monocyte numbers in 80- to 90-year-old people, there is an approximate tripling of monocyte numbers with age (Della Bella et al., 2007; Alpert et al., 2019). The relative proportion of non-classical and intermediate monocytes also increases with age, while classical monocytes decrease or remain unchanged (Nyugen et al., 2010; Seidler et al., 2010; Hearps et al., 2012; Figure 1). Without stimulation, a decrease in TNFα and IL-6 positive monocytes is seen with age (Nyugen et al., 2010). Upon stimulation of the toll-like receptors (TLRs), intermediate CD11c^+^ monocytes from aged individuals produce less inflammatory cytokines (IL-6 and TNFα) in response to TLR1/2 ligands, with no difference between young and old monocytes for TLR2/6 heterodimer (LTA), TLR4 (LPS), TLR5 (flagellin), and TLR7/8 (poly(U)) ligands (van Duin et al., 2007). Whereas classical monocytes respond similarly between young and old individuals following TLR4 stimulation, aged intermediated and non-classical monocytes had a greater increase in TNFα than the respective young monocyte populations (Hearps et al., 2012). While the magnitude of the initial response to TLR stimulation may be unaffected by age, aged murine macrophages have an impaired ability to end inflammatory responses (Pattabiraman et al., 2017).

Dendritic cells are the major antigen-presenting cells and play a critical role in bridging innate and adaptive immune responses. DCs can be divided into plasmacytoid DCs (pDCs), myeloid DCs (mDCs), and monocyte-derived DCs (MoDCs) (Figure 1C). Circulating pDC frequencies decrease with age, while the numbers of circulating mDCs and MoDCs are age-independent (Agrawal et al., 2017). However, DC phenotypes change with age. Unstimulated DCs from aged individuals have higher secretion of proinflammatory cytokines that likely contribute to age-related inflammation, or inflammaging (Figure 1D). DCs stimulated with LPS produce higher concentrations of proinflammatory cytokines and lower concentrations of an important anti-inflammatory cytokine, IL-10. For an excellent comprehensive review of DCs changes with age, see Agrawal et al. (2017).

### Adaptive Immune Cell Changes With Age

**FIGURE 2:**
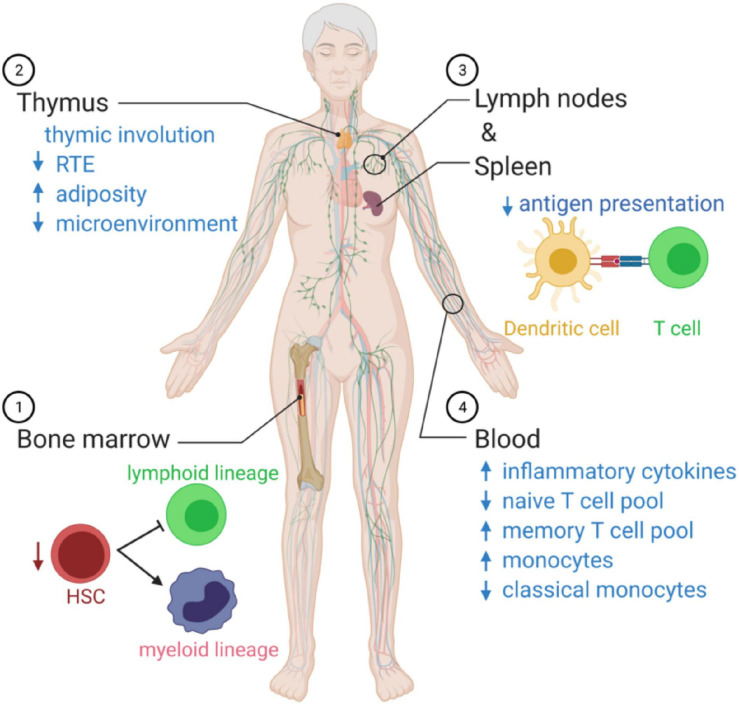
Summary of age-related changes in systemic immune function. (1) As we age, there is an imbalance in the production of innate immune cells and adaptive immune cells that favor cells of the myeloid lineage. (2) The microenvironment decreases with age, and there is a reduction in recent thymic emigrants (RTEs), reducing the naïve T cell poll in the circulation. (3) Antigen presentation by dendritic cells declines with age. (4) In the blood, the immunosenescence leads to a state of chronic inflammation, associated with elevated circulating cytokines, a decrease in naïve T cells able to respond to new pathogens, and monocytes over-produce inflammatory mediators and fail to resolve the inflammatory response.

Aging polarizes the immune system in favor of the innate immune response with a profound decline in adaptive immunity (Figure 2). The hematopoietic stem cell (HSC) pool declines with age and is skewed toward the production of myeloid cells (Dykstra et al., 2011). T cells’ progressive decline with age continues with lymphopoiesis and the age-related decline of thymic lymphoid progenitors that in turn reduce T cell generation (Berent-Maoz et al., 2012). The thymus is stable in size with age in humans, but the microenvironment is altered with adipose tissue replacing healthy tissue that supports T cell development (Velardi et al., 2020). As the output of naïve T cells from the thymus slows with age, the body switches to a peripheral proliferation of T cells to maintain the pool of naïve cells in circulation (den Braber et al., 2012).

Episomal DNA fragments are excised during T-cell receptor (TCR) gene rearrangement. These episomal DNA fragments, called TRECs, provide a direct estimate of *de novo* T cell generation, as they are not regenerated and are thereby diluted in dividing peripheral T cell (Kong et al., 1999; Hazenberg et al., 2000; Ribeiro and Perelson, 2007). By the age of 55 years old, it is estimated that only 5% of naïve T cells are produced in the thymus (Murray et al., 2003), and overall thymic output is decreased by more than 95% in 60-year-old people compared to 25-year-old people as measured by TREC (Naylor et al., 2005). Unlike mice, which produce most naïve T cells through thymic output, humans rely primarily (90% in young adults) on peripheral T cell proliferation (den Braber et al., 2012).

The decline in HSC polarization and thymic function with age is important following immunological insults such as infection and cancer when the immune system can be depleted in older individuals who cannot subsequently recover it (Velardi et al., 2020). Latent persistent infections are also part of the secondary changes leading to immunesenescence (Nikolich-Zugich, 2008).

Overall numbers of T cells in circulation are relatively unaffected by age, despite the decline in recent thymic output. However, there are changes in the distribution of different T cell subsets. The naïve T cells in older individuals have increased turnover, and virtual memory cells’ formation replaces the lost recent thymic emigrants. The virtual memory cells lose some of the naïve T properties, secrete Th1-type cytokines, and have decreased proliferation compared to naïve T cells (Nikolich-Zugich, 2014). Naïve CD8 T cells also decline, while naïve CD4 cells are more stable with age (Wertheimer et al., 2014). While the diversity of CD4 T cells is maintained through much of adulthood, a rapid decline in diversity was reported over the age of 70 years (Naylor et al., 2005). For instance, regulatory T cells (Tregs) and Th17 cells numbers decline with age (Vukmanovic-Stejic et al., 2011; Schmitt et al., 2013; Alpert et al., 2019). Moreover, there is a mismatch of cell number and cell function with age.

**FIGURE 3:**
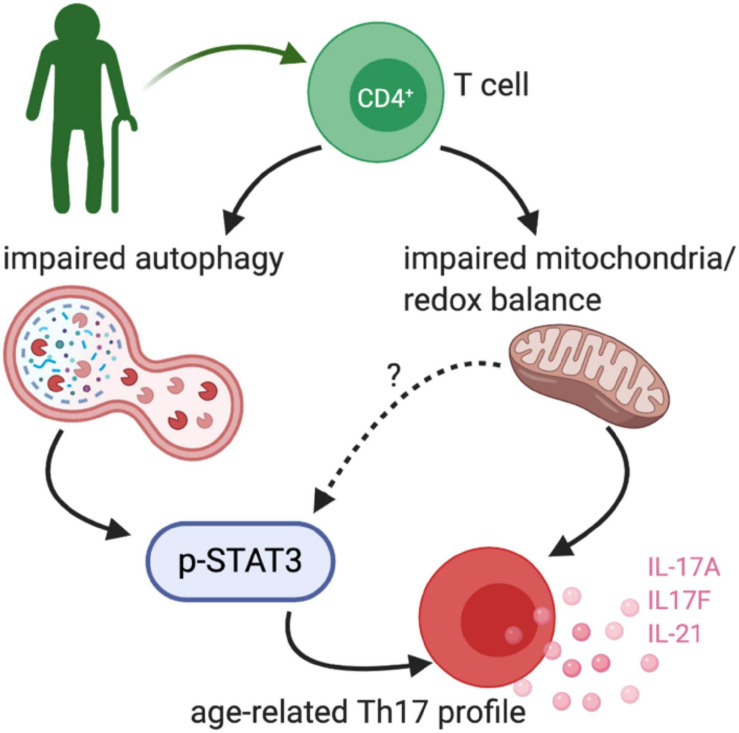
Age-related TH17 changes. Bharath et al. (2020) demonstrated that peripheral blood CD4+ cells from healthy older adults (∼60 years old) had an exaggerated TH17 profile. The TH17 signature was driven by impaired non-mitochondrial autophagy and dysfunctional mitochondria, at least in part via a STAT3 dependent mechanism.

Our recent work compared combinatorial cytokine profiles generated by CD4^+^ T cells from lean, normoglycemic older (average age ∼60) and younger (average age ∼32) subjects and concluded that Th17 cytokines dominate at least part of the inflammatory landscape in older subjects (Figure 3). The age-related Th17 profile is reminiscent of a type 2 diabetes-associated Th17 profile (Ip et al., 2016) and raises the possibility that age-related overexuberance of Th17s foster age-related declines in metabolic control. T cells from older compared to younger subjects had defects in autophagy and mitochondrial bioenergetics that associate with redox imbalance, and thus mirror age-related changes in non-immune cell types. The importance of autophagy defects in age-related inflammation, or inflammaging, were highlighted in cause/effect studies that impaired the autophagy machinery in T cells from young subjects with siRNA. This manipulation disrupted redox balance and activated Th17 profile cytokines in young cells by activating the Th17 master regulator STAT3, which in turn bound IL-17A and F promoters. Surprisingly, mitophagy-targeting siRNA failed to activate the Th17 profile in CD4^+^ T cells from young subjects, consistent with the idea that non-mitochondrial autophagy plays an independent role in age-related inflammation. Overall, our data support a model in which age-related changes in mitochondrial bioenergetics and non-mitochondrial autophagy converge to promote inflammaging (Bharath et al., 2020). The relationship between the age-associated CD4^+^ T cell profile and cognitive decline with age remain unexplored.

## Systemic Immune Changes Seen in People With Dementia

An ever-growing body of evidence shows interaction between the immune and nervous systems (Galea et al., 2007; Prinz and Priller, 2017). Studies dating back to the 1980s provided evidence that both the adaptive and innate immune cells are altered in people with dementia. This section describes changes in systemic immune cells highlighted by this work. The interested reader should also consider previous reviews on the topic (Rezai-Zadeh et al., 2009; Sommer et al., 2017) and reviews of systemic immune changes in animal models of neurodegenerative disease (Nevo et al., 2003; Cao and Zheng, 2018; Mayne et al., 2020).

In the 1980s, studies evaluated both changes in proportions of different immune cell populations (i.e., immunophenotyping) and how immune cells differed in response to a stimulus or mitogen (i.e., immune function). Over the subsequent 40 years, our understanding of the immune system has dramatically expanded. For example, we now have further divided T lymphocytes into smaller, specialized populations of cells using multi-color flow cytometry with parallel functional studies. Also, populations of cells such as “suppressor” T cells, which were dismissed in the late 1980s as artifacts, have now become identified as Tregs, arguably the most intensively studied CD4^+^ T cell subset in the last decade. We also know that there are many forms of dementia in the community, with a pure AD being relatively rare. So, when going back into the archives of older research, one must be aware of the limitations but also appreciate the strengths of these early studies.

### Whole Blood Cell Counts in AD and Related Dementias

One of the primary immunological changes seen with age in humans is the immune system’s polarization in favor of the innate immune response with a profound decline in adaptive immunity. Are further changes seen in the balance between innate and adaptive immunity in people with dementia? van der Willik et al. (2019) addressed this question in a powerful but simple way in the Rotterdam Study – a large prospective population-based cohort study of older adults (Ikram et al., 2017; van der Willik et al., 2019). As part of the Rotterdam study, van der Willik et al. (2019) evaluated blood cell counts in 8313 participants. Longitudinal evaluation of the complete blood counts was done for multiple visits (every 3–5 years) during a 13-year period. The study excluded people with dementia at the start of the study. Participants were screened for dementia at baseline, and each visit using the Mini-Mental State Examination (MMSE) and the Geriatric Mental Schedule score. During the follow-up period, 664 of the cognitively healthy individuals developed dementia, with 82% being probable AD-type dementia and 5% probable vascular dementia (VaD).

**FIGURE 4:**
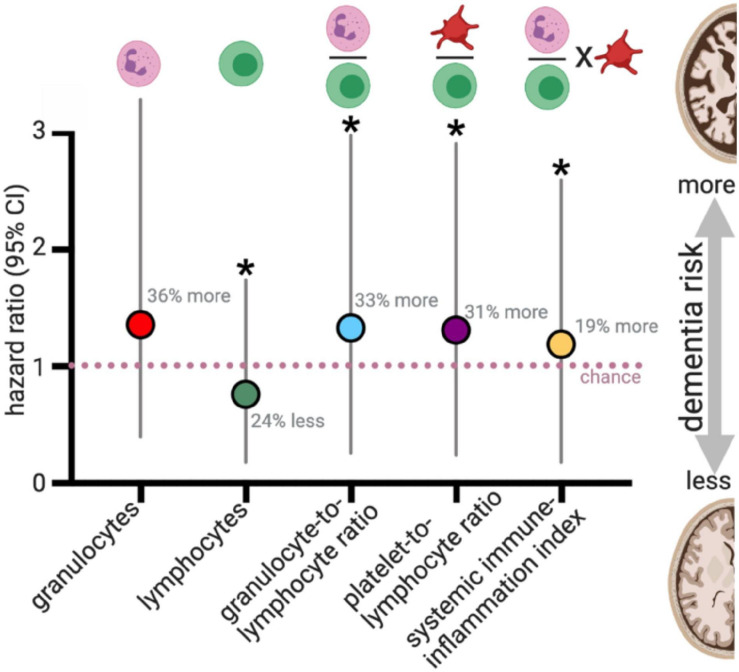
Blood cell counts and risk for all cause dementia. van der Willik et al. (2019) evaluated blood cell counts on 8313 participants who were dementia-free at the start of the study (mean age: 61 years old at the start). During a median follow-up of 8.6 years, 664 developed dementia. For those who did not have a stroke during the study (*N* = 8008), changes in blood cell counts, and the ratio of blood cell populations were statistically associated with dementia risk. Plotted results are from Table 3 of van der Willik et al. (2019). *p < 0.05.

To evaluate the balance of innate vs. adaptive immunity, they looked at ratios amongst blood granulocytes, platelets, and lymphocytes, which has recently been proposed to be a validated (albeit simple) measure of changes in immunity (Hu et al., 2014; Templeton et al., 2014a, b; Fest et al., 2018). Three predictive ratios were evaluated: (1) granulocytes-to-lymphocytes; (2) platelets-to-lymphocytes, and (3) a total systemic immune-inflammation index (integrated peripheral lymphocyte, neutrophil, and platelet indicator). van der Willik et al. (2019) found that altered balance between innate (granulocytes) and adaptive immunity (lymphocytes) was associated with an increased risk for developing all-cause dementia over those 13 years (median follow-up 8.6 years) (Figure 4). These results were consistent when correcting only for age and sex or adjusting the model by adding additional variables into the model (education, smoking status, body mass index, diabetes mellitus, history of stroke, and APOE4 carriers). The immune cell balance changes were informative, suggesting that dementia was exacerbating the normal age-related changes in the immune balance – more innate granulocytes and fewer adaptive immune cells. VaD showed greater hazard ratio changes for increased innate activity (granulocyte number, and granulocyte-to-lymphocyte ratio). VaD associated with lower indicators of adaptive immunity (lymphocyte number, and platelet-to-lymphocyte ratio) in comparison to all-cause dementia and AD. These results are in general agreement with four earlier studies that evaluated blood count changes in people with dementia, which found a decrease in lymphocyte number and increase in neutrophil number in the AD vs. the aged-matched control groups (Tollefson et al., 1989; Kalelioglu et al., 2017), and increased neutrophil-to-lymphocyte ratios (Kuyumcu et al., 2012; Rembach et al., 2014; Kalelioglu et al., 2017). In contrast, one study found no difference in lymphocytes between AD and control participants (Dysken et al., 1992). Differences in study design (cross-sectional), smaller sample size, age at the start of the study, and covariate adjustment should all be considered when comparing the results from earlier studies that disagree with van der Willik et al. (2019). Nevertheless, these studies provide compelling evidence for dementia-specific disruption of the immune system atop shifts that characterize healthy aging.

### T Cell Immune Phenotyping in AD and Related Dementias

Leffell et al. (1985) were among the earliest studies to complete immune profiling in people with AD. Their cross-sectional study found no change in total T cells, CD4, CD8, or the ratio of CD4:CD8 cells between the people with probable AD-type dementia and aged-matched controls. In their concluding paragraph they stated, “we believe that there is little, if any, cumulative evidence to support an immunological process in Alzheimer’s disease.” However, this statement needs context, as they, and many studies that followed, explored if AD was caused by an autoimmune attack on neurons similar to multiple sclerosis, or by infectious agents like prions in Creutzfeldt–Jakob disease.

In the past 25 years, changes in the major T cell populations have been evaluated across different stages of dementia progression, spanning from mild cognitive impairment (MCI) to severe AD (Table 1). Throughout the late 1980s and 1990s, a handful of studies evaluated the immune system in people with dementia (Singh et al., 1987; Antonaci et al., 1990; Licastro et al., 1990; Araga et al., 1991; Ikeda et al., 1991; Pirttila et al., 1992; Singh, 1994; Hu et al., 1995; Shalit et al., 1995; Trieb et al., 1996; Lombardi et al., 1999). The majority of studies found no change at any stage of disease progression for total T cells (CD3), or CD4 or CD8 T cells. Some of the studies found no statistical relationship between dementia and immunological functions. For instance, IL-2 was equally effective at inducing proliferation of lymphocytes in AD and control participants (Licastro et al., 1990). While a few studies found no change in CD4, CD8 or CD4:CD8 ratios (Ikeda et al., 1991; Singh, 1994), others found changes in these cell populations (Pirttila et al., 1992). The changes include a decrease in CD8 cells in probable AD dementia and an increase in CD8 cells in probable VaD both compared to age-matched controls (Pirttila et al., 1992; Hu et al., 1995; Shalit et al., 1995). Shalit et al. (1995) found that the severity of the neurodegenerative disease correlated with the immune cell changes including an increase in CD4s and a decrease in CD8s in severe dementia, despite no differences in CD4 or CD8 numbers in mild dementia, both compared to controls. Tregs were found to change by dementia status; however, future studies are needed to replicate these studies using state of the art markers for human Treg cells. A single recent report found that TH17 cells were increased in MCI, but by early AD the increase in TH17 cells was no longer apparent (Oberstein et al., 2018). It will be necessary to replicate these changes in future studies and evaluate these changes in a longitudinal instead of a cross-sectional study.

**TABLE 1:** Relationship between dementia status and T cell populations.

	CD3	CD4	CD8	CD4/CD8	Treg	TH17
MCI vs. aged-matched control	⟷ (Lueg et al., 2015)	⟷ (Lueg et al., 2015)	⟷ (Lueg et al., 2015), ↑ (Gate et al., 2020)	⟷ (Lueg et al., 2015), ↓ (Gate et al., 2020)	⟷ (Le Page et al., 2017), ⟷ (Oberstein et al., 2018), ↑ (Saresella et al., 2010)	↑ (Oberstein et al., 2018)

Early AD vs. aged-matched control	⟷ (Lueg et al., 2015)	⟷ (Lueg et al., 2015), ⟷ (Bonotis et al., 2008), ⟷ (Shalit et al., 1995)	⟷ (Shalit et al., 1995), ⟷ (Lueg et al., 2015)	⟷ (Lueg et al., 2015)	⟷ (Le Page et al., 2017), ⟷ (Oberstein et al., 2018), ↑ (Saresella et al., 2010)	⟷ (Oberstein et al., 2018)

Late AD vs. aged-matched control	⟷ (Busse et al., 2017), ↓ (Richartz-Salzburger et al., 2007), ⟷ (Pirttila et al., 1992)	↓ (Bonotis et al., 2008), ⟷ (Richartz-Salzburger et al., 2007), ↑ (Shalit et al., 1995), ⟷ (Pirttila et al., 1992), ⟷ (Leffell et al., 1985)	⟷ (Bonotis et al., 2008), ⟷ (Richartz-Salzburger et al., 2007), ↓ (Shalit et al., 1995), ↓ (Pirttila et al., 1992), ⟷ (Leffell et al., 1985)	⟷ (Bonotis et al., 2008), ⟷ (Richartz-Salzburger et al., 2007), ↑ (Shalit et al., 1995), ↑ (Pirttila et al., 1992), ⟷ (Leffell et al., 1985)	⟷ (Busse et al., 2017), ⟷ (Rosenkranz et al., 2007)	

Early AD vs. MCI	⟷ (Lueg et al., 2015)	⟷ (Lueg et al., 2015)	⟷ (Lueg et al., 2015)	⟷ (Lueg et al., 2015)	⟷ (Le Page et al., 2017), ⟷ (Saresella et al., 2010)	

Late AD vs. early AD		↓ (Bonotis et al., 2008)	⟷ (Bonotis et al., 2008)	⟷ (Bonotis et al., 2008)		

AD vs. other dementia	⟷ (Lueg et al., 2015), ⟷ (Busse et al., 2017)	⟷ (Lueg et al., 2015), ⟷ (Busse et al., 2017)	⟷ (Lueg et al., 2015), ⟷ (Busse et al., 2017)	⟷ (Lueg et al., 2015)	⟷ (Busse et al., 2017), ⟷ (Rosenkranz et al., 2007)	

AD vs. VaD	⟷ (Busse et al., 2017)	⟷ (Busse et al., 2017)	⟷ (Busse et al., 2017)		⟷ (Busse et al., 2017)	

*The symbols indicate the following: ⟷ no change, ↑ increase in cell population, and ↓ decrease in cell population. MCI (mild cognitive) impairment (CDR 0.5), Early AD (CDR 1–2), and Late AD (CDR 3). If the clinical dementia rating (CDR) scale or MMSE was not specifically noted in the paper, the cases were assumed to be LATE AD.*

**FIGURE 5:**
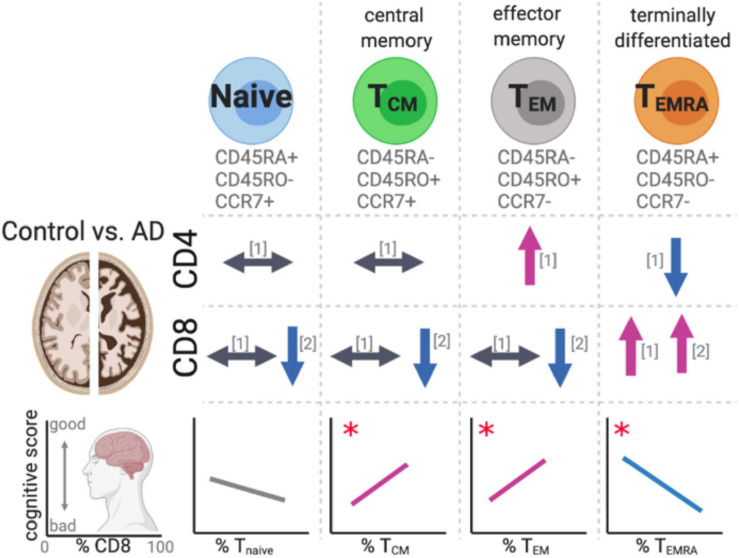
Changes in T cell subsets in AD. Recent studies have identified changes in effector memory and terminally differentiated T cells in AD patients’ peripheral blood compared to aged-matched controls. The changes in the T cells subsets were found to significantly correlate with cognitive scores. References for changes correspond to the following: [1] (Busse et al., 2017) and [2] (Gate et al., 2020). The symbols indicate the following: ⟷ no change, ↑ increase in the cell population, ↓ decrease in the cell population in AD cases compared to aged-matched controls. *p < 0.05.

Two recent studies have evaluated naïve, central memory (T_*CM*_), effector memory (T_*EM*_), and terminally differentiated (T_*EMRA*_) CD4 and CD8 subsets (Busse et al., 2017; Gate et al., 2020; Figure 5). The four subsets of T cells were identified by the expression of lymph node homing molecule receptor, CCR7. Along with the expression pattern of the leukocyte common antigen (CD45) isoforms RA and RO. During the transition from naïve to T_*EM*_ the expression of CCR7 is lost when the T_*CM*_ are restimulated by antigen. When the T_*EM*_ terminally differentiate, they stop expressing CD45RO and re-express CD45RA. It was found that the CD8^+^ T_*EMRA*_ were increased in the blood of MCI/AD patients. The percentage of these CD8^+^ T_*EMRA*_ cells were strongly correlated with worse cognitive function (Gate et al., 2020). In the AD patient’s cerebrospinal fluid, clonal expansion of the CD8^+^ T_*EMRA*_ was found. While the authors identified TCRβ sequence with known specificity for the *Herpesviridae* Epstein–Barr nuclear antigen 3, they cautioned against suggesting that these finding provided evidence for a link between Epstein–Barr virus and AD, as it has infected up to 95% of American adults, and their caution is well founded.

### T Cell Immune Function in AD and Related Dementias

Skias et al. (1985) used immunopanning to enrich for CD4 and CD8 cells from peripheral blood mononuclear cells (PBMCs) to evaluate function of the T cells. In the cross-sectional design they included 16 AD (mean age 75) and 14 age-matched controls (Skias et al., 1985). They found in the CD8 enriched cell fraction that upon stimulation with pokeweed mitogen the CD8 cells from AD participants were less able to suppress IgG production from B cells. Skias et al. (1985) postulated that the changes in immune function in AD could be a form of “accelerated aging” and that the “selective neuronal loss could lead to perturbed neural influences on the immune system.”

Miller et al. (1981) also found that lymphocytes from AD participants compared to aged-matched controls (mean age 75.5) had less response to the mitogen phytohemagglutinin, indicating an overall blunted immune response. They also reported increased immune suppression activity in the AD lymphocytes in response to another non-specific mitogen, concanavalin-A, compared to lymphocytes from age-matched controls. Miller et al. (1981) had a third view on the immune changes in AD. They speculated that AD might be more like a chronic viral infection in the way that it suppresses immunity. Further, they speculated that the “active impairment of a particular immune response has allowed an infectious agent to gain a foot in [dementia patients], resulting in central nervous system damage.” While these findings are 40 years old, and could be more compelling, the ideas proposed are interesting and should be revisited.

Trieb et al. (1996) tested if PBMCs from AD cases would respond to amyloid precursor protein (APP) fragments (Aβ1–28). The study included 20 old healthy controls (mean age 71), 20 young healthy controls (mean age 25), and 10 participants with AD (moderate severity *n* = 8 and severe *n* = 2; mean age 73). They found that the AD PMBCs had a decreased response to Aβ1–28 compared to the young or old controls as measured by a proliferation assay, with no effect of age. While it could be speculated that this was some sort of tolerance to Aβ stimulation in AD cases, the effects could also be from a general lack of effector function in PMBC from individuals with AD. Subsequent studies found no difference in the ability of PBMCs to respond to Aβ1–42 between AD vs. control participants (Giubilei et al., 2003; Rocha et al., 2012).

**FIGURE 6:**
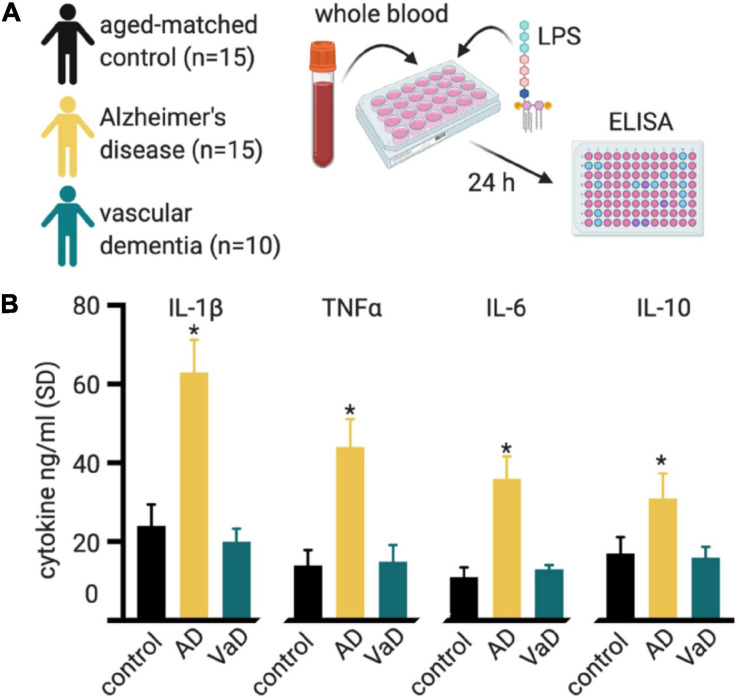
Whole blood cultures show an exaggerated response to LPS. **(A)** Lombardi et al. (1999) stimulated whole blood cultures from individuals with Alzheimer’s disease (AD), vascular dementia (VaD), and compared these to aged-matched controls. Cell culture supernatant was collected at 24-h intervals for 72 h for ELISA cytokine assays. **(B)** All cytokines measured showed higher cytokine production in the AD group, compared to the control or VaD groups. The plotted data is from the 24 h post-stimulation time point, from Table 3 of Lombardi et al. (1999). A similar trend was seen for the 48 and 72 h post-stimulation timepoint. *p < 0.05 compared to control.

Singh (1994) stimulated PBMCs from probable AD cases or controls with LPS, which activates mainly innate immune cells through TLR4. They found that not only was there a bias toward increased innate immune cell numbers, but those innate immune cells produced 2.3-fold more IL-1 and threefold more IL-6 compared to age-matched control PBMCs (Singh, 1994). Lombardi et al. (1999) expanded upon these findings by measuring a more comprehensive panel of cytokines and including VaD and Parkinson’s disease groups. They found that LPS stimulated whole blood cultures from AD patients showed differences in cytokine production compared to results from aged-match controls and the VaD and Parkinson’s disease groups (Figure 6; Lombardi et al., 1999). The results support the case for an exaggerated innate immune response and also suggest signatures of immune function that are unique to different forms of dementia.

By the age of 40, individuals with Down’s syndrome will have AD-neuropathological changes in their brains, and the majority will develop dementia. Park et al. (2000) isolated peripheral blood monocytes from individuals (range 26–68 years old, mean 45 years old) with Down syndrome and age-matched controls and stimulated the cells with LPS. They found no difference in TNFα, IL-6, or IL-8. In contrast to Singh (1994) and Lombardi et al. (1999), they found that monocytes from Down’s syndrome individuals produced 30% less IL-1β than the aged match controls.

Stimulating PBMCs from AD patients or aged-matched controls with either PHA or anti-CD3/CD28 it was found that the AD patients had an increased proinflammatory IL-1β response, presumably from the innate immune cells, a decreased in the anti-inflammatory cytokine IL-4, presumably from T cells (Reale et al., 2004; Rocha et al., 2012). Stimulating the PBMCs with PHA-M, a T cell activator, IL-2 was increased in the late AD, but not in the early AD compared to control (Shalit et al., 1995). AD patient CD8 T cells stimulated with PMA and ionomycin produced more IFNγ and TNFα than CD8 T cells from age-matched controls (Gate et al., 2020). In summary, both innate and adaptive immune cells’ products upon activation are dysregulated in people with dementia. More work is needed to understand the inflammatory signature of this dysregulation and the role of immune cell cross-talk, as these areas will be important targets for therapeutic intervention.

### B Cell Changes in AD and Related Dementias

Despite the emerging understanding of roles for T cells in AD and related dementias, few studies explored roles for B cells in AD, although B cells coordinate with T cells to define functions of the adaptive immune system. B cells can proliferate in T cell-dependent manner or independent manner with the latter producing immunoglobulins (Ig), also termed antibodies, with low affinity and failure of memory B cell development (Defrance et al., 2011). B cell proliferation in a T cell-dependent manner is facilitated by helper T cells after antigen-presentation to give rise to memory. In addition to producing Ig, B cells act as antigen presenting cells to induce T cell activation through co-stimulation and cytokine production (Chalasani and Rothstein, 2014).

Age-related diseases like chronic lymphocytic leukemia and autoimmune diseases show extremes of B cells changes with age (Candore et al., 1997; Chiorazzi and Ferrarini, 2003). While there is a decrease in total B cells in the elderly, age-related increases in particular B cell populations may be involved in increased inflammation (Colonna-Romano et al., 2009; Bulati et al., 2014). Unfortunately, only a few reports describe B cell status in AD and related dementias. IgG^+^IgD^–^CD27^–^ B cells are increased in peripheral blood of patients with moderate to severe AD, compared patients with mild AD or no cognitive impairment (Bulati et al., 2015). B1 cells, which make IgM and are considered more “innate-like” in function, have been implicated in amyloid-beta clearance in people (Agrawal et al., 2018) and mice (Baulch et al., 2020): both B1 and IgM are decreased in an AD-relevant mouse model (Baulch et al., 2020). Using an *in vitro* model with B cells from AD patients further suggests a role for B cells in amyloid-beta plaque formation (Dezfulian, 2018). Despite the paucity of studies, the available evidence supports the future exploration of B cell function in AD and related dementias.

## Conclusion and Future Directions

Aging causes a functional decline and reorganization of the immune system collectively termed immunesenescence, which leads to a chronic state of inflammation that damages many organs, including possibly the brain. Chronic systemic inflammation is greater in people with AD (Holmes et al., 2009). Immunesenescence leaves the body vulnerable to infections and suboptimal vaccine responses through poorly understood mechanisms and leaves the immune system at the risk of being depleted following an infection or injury. In people with AD dementia, and perhaps other dementias, the age-related change in immune system cell distribution and function is exacerbated. The balance of innate vs. adaptive immune systems is further skewed toward innate immune function in people with AD (van der Willik et al., 2019). The decrease in memory T cells and increase in clonal expansion of T_*EMRA*_ T cells tracks closely with decline in cognitive function in AD (Gate et al., 2020). When the systemic immune cells encounter stimuli they are primed toward an exaggerated proinflammatory response (Singh, 1994; Godbout et al., 2008).

In the neuro-centric view, AD starts in the brain. This could be, as proposed by Leffell et al. (1985), that AD is caused either by a neuron-targeted autoimmune attack or by an infection in the brain that damages the neurons, and that the immune system is altered in response to this infection. Many infectious agents have been proposed over the years (Readhead et al., 2018; Drayman et al., 2019; Abbott, 2020; Allnutt et al., 2020; Gate et al., 2020; Rizzo, 2020). The alternative neuro-centric view is that as neurons are degenerating, there is a loss of neural trophic support, which suppresses the immune system, as postulated by Skias et al. (1985). However, while both of these views have merit, we believe that the current evidence does not support these interpretations.

From the immunological centric standpoint, immunosenescence comes first. As the immune system becomes dysfunctional, we speculate that it is no longer able to protect the brain. The genetic evidence strongly supports the role of the immune system in AD (Kunkle et al., 2019; Andrews et al., 2020; Neuner et al., 2020), as many genetic risk variants associate with innate immunity. Could these genetic risk variants further drive the age-related imbalance in adaptive vs. innate immunity? Or are genetics additive to the effects of aging – that is, more innate cells that are more proinflammatory? Currently, the vast majority of work on innate immune cells in AD is focused on the microglia. Comparatively, much less is known about how AD risk genes affect the systemic innate immune function, and more work is needed in this area.

While it is often assumed that the decline in dementia is steady and progressive, at the individual level, the decline is more zigzag with troughs of decline, which are speculated to correspond to life events (infections, diseases, surgeries, etc.) (Perry et al., 2007). If a dysfunctional immune system leads to more numerous/serious infections, or exaggerated responses to the infection, could these events be driving a neurodegenerative decline? One could speculate that it is not until that immune system is challenged (for example, by a urinary tract infection) that an exaggerated immune response damages the brain. However, more work is needed to test these possibilities.

**FIGURE 7:**
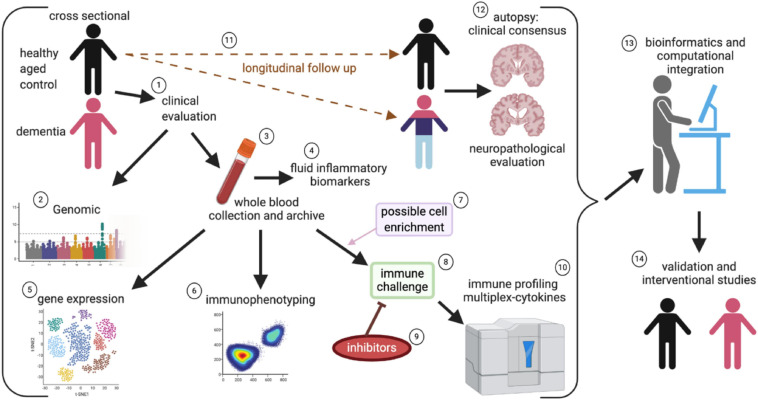
Systems approach to evaluate systemic immune changes in dementia. In a community-based cohort of individuals who are followed longitudinally and have agreed to brain autopsy and donation, the study can begin with a cross-sectional design of participants without cognitive impairment and comparing those with mild cognitive impairment to profound dementia, and incorporating clinical evaluations as part of the study (1). By piggybacking on a larger study that incorporates genomic (2), blood collection (3), and fluid biomarker assessment (including assays for systemic inflammation), (4) it is possible to leverage ongoing cohort studies to better define the role of immune dysfunction in AD. Peripheral blood mononuclear cells (PBMC) are isolated from the fresh whole blood and are archived for future studies (3). The isolated PMBCs can be used for gene expression and gene sequencing to identify changes in immune cell populations and clonal expansion of T cells (5). Immunophenotyping of immune cell subsets can be done to evaluate changes in a population of immune cells (6). Select populations of immune cells can be enriched (7). The PBMC or select cells can be directly stimulated with activators such as LPS or CD3/CD28 (8), to evaluate cellular mechanisms and therapeutic targets, inhibitors can be added along with the mitogens (9). The cell culture supernatant can then be used for cytokines multiplex immune profiling assays (10). By enriching the cross-sectional design for healthy controls, it is possible to evaluate people’s possible conversion into varying neurodegenerative diseases (11), which are ultimately pathologically confirmed at autopsy (12). The wealth of data that is captured will require a very robust statistical and bioinformatical infrastructure to integrate all the data (13). Finally, “hits” will require validation in a subsequent set of study participants, and clinical experimentation can test positive intervention strategies targeted at restoring the immune balance (14).

In conclusion, there is compelling correlational evidence that the systemic immune system is disrupted in people with dementia. There is also strong evidence that genetic polymorphisms in immune function, including HLA and TREM2, modify the risk for developing AD. Still, there are several essential questions. Our review provides compelling evidence that the immune system is altered in AD, but our current knowledge of how the immune system is changed is fragmented. Future studies (Figure 7) are needed to provide a complete picture of how the immune system changes in people with healthy cognitive aging and those who transition into neurodegenerative disease. Such studies are costly, but by partnering and incorporating immunological evaluations as part of ongoing analysis of cognitive state in community cohorts (Schmitt et al., 2012), we can address many unresolved questions in the field.

In addition to understanding the natural history of immune changes associated with AD and related dementias, it will also be important to establish causation, mechanisms of dysfunction, and ultimately therapies. Future studies will require a translational approach using animal models. Example studies from animal models show the influential role of the adaptive immune system as a modifier of brain function. For example, dietary and metabolic manipulations have been shown to lead to an increase in Th17s and Th17-cytokines (IL-17, IL-22) that cause cognitive dysfunction in the absence of amyloid plaques and neurofibrillary tangles (Faraco et al., 2018). Alternatively, boosting adaptive immune system function through blockade of immune checkpoints, such as PD-1, improved outcomes in multiple AD-relevant animal models (Baruch et al., 2015, 2016; Rosenzweig et al., 2019). In contrast, in Rag2, Il2rγ, double-knockout mice that lack T cells, B cells, and natural killer cells, the lack of an adaptive immune system worsens pathology and neuroinflammation in an AD-relevant mouse model (Marsh et al., 2016). These animal studies and others suggest that the adaptive immune system contributes to cognitive decline (Spani et al., 2015; Jevtic et al., 2017; Unger et al., 2020). This interpretation is therapeutically promising as it provides opportunities for manipulation, including approaches such as IL-7 to replenish the depleted immune system and checkpoint inhibitors like PD-1. Much more work is still needed. By gaining a better understanding of the connection between the immune system and the brain there is great promise in obtaining a more holistic perspective of how the demise of mind and body both contribute to dementing diseases.

## Author Contributions

AB completed the literature review and wrote the first draft. JL and BN wrote sections, compiled, contributed to the design and conceptualization of the study, and revising the manuscript for intellectual content. All authors read and approved the final manuscript.

## Conflict of Interest

The authors declare that the research was conducted in the absence of any commercial or financial relationships that could be construed as a potential conflict of interest.
